# Association of the SNP rs112369934 near *TRIM66* Gene with POAG Endophenotypes in African Americans

**DOI:** 10.3390/genes12091420

**Published:** 2021-09-15

**Authors:** Claire D. Kim, Harini V. Gudiseva, Brendan McGeehan, Ebenezer Daniel, Gui Shuang Ying, Venkata R. M. Chavali, Joan M. O’Brien

**Affiliations:** 1 Perelman School of Medicine, University of Pennsylvania, Philadelphia, PA 19104, USA; claire.kim@pennmedicine.upenn.edu; 2 Scheie Eye Institute, University of Pennsylvania, Philadelphia, PA 19104, USA; gudiseva@pennmedicine.upenn.edu (H.V.G.); brenmc@pennmedicine.upenn.edu (B.M.); ebdaniel@pennmedicine.upenn.edu (E.D.); gsying@pennmedicine.upenn.edu (G.S.Y.); vchavali@pennmedicine.upenn.edu (V.R.M.C.)

**Keywords:** glaucoma, primary open-angle glaucoma, genetic diseases, *TRIM66*, retinal nerve fiber layer, cup-to-disc ratio, African Americans

## Abstract

We investigated the association of the single nucleotide polymorphism (SNP) rs112369934 near the *TRIM66* gene with qualitative and quantitative phenotypes of primary open-angle glaucoma (POAG) in African Americans (AA). AA subjects over 35 years old were recruited for the Primary Open-Angle African American Glaucoma Genetics (POAAGG) study in Philadelphia, PA. Glaucoma cases were evaluated for phenotypes associated with POAG pathogenesis, and the associations between rs112369934 and phenotypes were investigated by logistic regression analysis and in gender-stratified case cohorts: The SNP rs112369934 was found to have a suggestive association with retinal nerve fiber layer (RNFL) thickness and cup-to-disc ratio (CDR) in 1087 male AA POAG cases, individuals with the TC genotype having thinner RNFL (95% CI 0.85 to 6.61, *p* = 0.01) and larger CDR (95% CI −0.07 to −0.01, *p* = 0.02) than those with wildtype TT. No other significant associations were found. In conclusion SNP rs112369934 may play a role in POAG pathogenesis in male AA individuals. However, this SNP has been implicated in higher POAG risk in both male and female AA POAG cases.

## 1. Introduction

Glaucoma is the leading cause of irreversible blindness worldwide, with approximately 3.6 million patients over the age of 50 years losing vision from this disease annually as of 2020 [1]. Glaucoma is characterized by the progressive degeneration and deterioration of the optic nerve head (ONH) and retinal nerve fiber layer (RNFL), with a corresponding loss of the visual field [2]. Most glaucoma patients are asymptomatic and unaware when irreversible vision loss occurs [3].

Primary open-angle glaucoma (POAG) features a trabecular meshwork (TM) outflow pathway for the aqueous humor that is accessible but can be blocked internally [2]. POAG more severely and more commonly affects patients of African ancestry [4]. In 2020, POAG cases in the adult population was estimated to be 52.68 million, with an anticipated increase to 79.76 million by 2040 [4].

Individuals of African ancestry comprise a disproportionately large percentage of POAG cases [3] and are four to five times more likely to be diagnosed with POAG than individuals with European ancestry [5], be younger at age of diagnosis, and are up to 15 times more likely to experience vision loss from the disease [6,7,8].

The etiology, pathophysiology, and progression of POAG are not well-understood. The known risk factors include older age [8], male sex [3], and positive family history [9]; ocular factors such as high IOP [10], disc hemorrhages [11], thin central cornea [12], and severe myopia [13]; and systemic conditions such as hypertension [14], type 2 diabetes mellitus [15], and low cerebral fluid pressure [16]. Despite being managed with IOP lowering medications or surgical procedures, many patients inevitably encounter progressive and permanent visual field (VF) deficits [17].

Many twin and family linkage studies demonstrate a strong genetic component to POAG [18,19,20,21], but much of the genetic components behind POAG pathogenesis remain unexplained [22]. In recent years, genome-wide association studies (GWAS) have revealed several significant variants in single nucleotide polymorphisms (SNPs) with small individual effects on the progression of POAG [23] and quantitative trait associations in genes such as MYOC, OPTN, WDR36, CDK2N2B-ASI, TMC01, XIS6, ABCA1, GAS7, FOXC1, NTF4, ASB10, EFEMP1, and IL20RB [22,24,25,26]. Furthermore, these variants have been associated with specific endophenotypes within POAG’s broad range of trait expression. Studying these variants can help identify higher-risk subgroups and improve the existing models that predict disease development and prognosis [27,28]. However, most of these variants were discovered and found to be significant in European and Asian populations with limited or unknown significance in African Americans [29,30,31]. To date, several studies such as the Genetic Epidemiology Research in Adult Health and Aging (GERA) cohort and the African Descent and Glaucoma Evaluation Study (ADAGES) have identified novel variants in individuals of African ancestry [32,33]. However, they did not investigate variants for POAG in the more admixed AA population.

Our team previously performed a large-scale GWAS project titled the Primary Open-Angle African American Glaucoma Genetics (POAAGG) study. This study recruited over 10,200 AA individuals from Philadelphia, Pennsylvania, to investigate POAG and identify potential variants specific to the AA population [34]. Case-control analysis and single SNP association analysis implicated a novel SNP on chromosome 11 (rs112369934) near the *TRIM66* gene [34], which belongs to the tripartite motif (TRIM) family of genes. The TRIM family codes for a group of well-conserved proteins that have been shown in transcriptome analysis to be one of the most highly expressed gene families in retinal progenitor cells (RPCs) of the developing mouse retina [35]. Prior studies from our team have shown that there is increased expression of *TRIM66* in induced pluripotent stem-cell-derived retinal ganglion cell cultures and trabecular meshwork cells in conditions of oxidative stress compared to control cells [34]. Given *TRIM66*’s implication in homeostasis management during stress conditions, we aimed to study *TRIM66* in POAG patients to further elucidate the role of this novel variant in POAG pathogenesis. In this study, we investigated whether the SNP rs112369934 near *TRIM66* is associated with POAG quantitative and qualitative features in our AA cohort.

## 2. Materials and Methods

### 2.1. Patient Demographics

Subjects were identified and characterized for the POAAGG study as previously reported [36]. In brief, subjects over 35 years of age and self-reported as Black, African American, or having African ancestry were considered. Subjects were recruited from all comprehensive and subspecialty ophthalmology clinics at the University of Pennsylvania and at external ophthalmology clinics in Philadelphia (Windell Murphy, MD; Temple University). Informed consent was obtained from all participating subjects. Approval for this project was obtained from the University of Pennsylvania Institutional Review Board, and research was performed according to the principles of the Declaration of Helsinki.

### 2.2. Phenotypes Collected

All eligible patients underwent an onsite ophthalmic examination and interview. POAG cases were defined as having an open angle with glaucomatous changes to the ONH in one or both eyes, characteristic VF deficits on two consecutive and reliable VF tests representing ON defects, and without secondary etiologies of glaucoma. The following quantitative phenotypic measures were obtained from each POAG case: central corneal thickness (CCT), cup-to-disc ratio (CDR), IOP, mean deviation (MD), pattern standard deviation (PSD), retinal nerve fiber layer (RNFL), and visual acuity (VA). Characteristics and distribution of available quantitative trait phenotypes are as previously described [34].

Qualitative phenotypes were obtained using a standardized grading form for 30-degree color stereo disc photos of study subjects, imaged with the Topcon TRC 50EX retinal camera (Topcon Corporation of America, Paramus, NJ, USA). Two non-physician graders were trained by glaucoma specialists to independently complete a standardized form for each pair of color stereo images [37]. Patients were excluded from analysis if they did not have disc photos or if the photos were deemed too low in quality. The optic nerve characteristics were categorized into disc/cup characteristics, peripapillary atrophy characteristics, and features of glaucomatous optic nerve head such as optic disc shape, optic disc size, cup shape, cup depth, presence and location of sloping adjacent to outer optic disc rim, presence and borders of peripapillary atrophy (PPA), presence of heavy pigmentation, disc hemorrhage, notching, pallor of optic disc, and disc tilt.

Characteristics of the qualitative phenotypes are described as follows:

1. Disc shape: shape of the disc only without the surrounding PPA. Discs were categorized as round or oval;
2. Shape of cup: cup shape was described as conical, cylindrical, or bean-pot shaped (excavated cup);
3. Cup depth: depth of cup was described as shallow, moderate, or severe;
4. Rim plane position: neuroretinal rim was evaluated to see if any portion of the rim was at a lower plane than other parts of the rim;
5. PPA: each image was assessed for presence of β PPA;
6. Disc hemorrhage: defined as isolated flame-shaped or splinter-like hemorrhages on the disc or in the immediate peripapillary area;
7. Notching of the cup;
8. Pallor of optic disc: presence of diffuse or localized disc pallor was recorded.

### 2.3. Genotyping

Specimen collection, genotyping, and quality control were performed as previously described in the POAAGG study [34]. Genomic DNA was isolated and sequenced from blood and/or saliva samples. Blood was collected and DNA precipitated from blood samples using Gentra PureGene kits (Qiagen, Valencia, CA, USA). Saliva was collected from subjects via Oragene DISCOVER (ORG-500) self-collection kits (DNA, Genotek, Canada). After collection, isolation, and purification, DNA concentrations were confirmed with the fluorescence-based Quant iT dsDNA Board-Range Assay kit (cat # Q33130, Life Technologies, CA, USA) using a Tecan Infinite M 200 Pro multimode microplate reader (Tecan, Morrisville, NC, USA).

Furthermore, 2559 DNA samples from cases were successfully genotyped using the Multi-Ethnic Genotyping Array (MEGA) V2 (EX) consortium chip on the Infinium iSelect platform (Illumina, San Diego, CA, USA). The SNP near the *TRIM66* is extracted from the genotyping data previously generated as described in POAAGG GWAS [34].

### 2.4. Statistical Analysis

Measurements closest to the study enrollment date were chosen as the baseline measures for statistical analysis. For the continuous clinical phenotype measurements, the descriptive means and standard deviations (SD) by genotype groups were reported. For categorical phenotype measurements, the counts and percentages were reported. For the analysis of rs112369934 among glaucoma cases, only six subjects had the CC genotype; these patients were excluded from the statistical comparisons of phenotype measurements for SNP rs112369934 genotypes (e.g., TT vs. TC). Comparisons of the baseline ocular clinical phenotype and optic disc parameters between the genotype groups (e.g., TT vs. TC) were performed using generalized linear models, and the inter-eye correlation was accounted for by using estimating equations (GEE) [38,39]. In the GEE, an independent working correlation structure was specified to describe the correlation in phenotype measurements between two eyes from the same patient. Data from each patient were identified to the computing algorithm by specifying a unique identification number for each patient. For patients with phenotype measurements on both eyes, the correlation between eyes was involved in the calculation of standard errors. From these models, the mean difference and 95% confidence interval (95% CI) were obtained for continuous measurements, and corresponding *p*-values are reported for both continuous and categorical measures. These analyses were performed for all POAG cases together, and by males and females separately because their associations may differ by gender. All the significant *p*-values were evaluated using Bonferroni correction method (i.e., corrected *p*-value was calculated as 0.05 divided by number of phenotypes evaluated). All analyses were performed in R version 4.03 (Revolution Analytics, Dallas, TX, USA).

## 3. Results

### 3.1. Association of SNP rs112369934 with Quantitative or Qualitative Phenotypes

The present association study includes 2559 POAG cases with 3689 eyes. Quantitative and qualitative phenotypic data were not collected for the POAAGG study control group, and thus the analysis for SNP association with phenotypes was performed for POAG cases only. The patients had a mean age of 69.8 years and included 1087 males and 1472 females. The males have 11.3% frequency of TC genotypes while females have 13.9%.

The statistical analysis was performed to investigate associations between SNP rs112369934 and each qualitative and quantitative phenotype. In the quantitative phenotypes, there were no significant associations detected between the TC variant and the endophenotypes (Table 1, all *p* > 0.05).

Among the qualitative phenotypes, there were no significant associations detected (Table 2, all *p* > 0.08). The presence of optic disc tilting was near statistical significance, with TC variants at SNP rs112369934 more likely to have disc tilt (8.8% vs. 5.4%, *p* = 0.08). Other anatomical characteristics of the posterior eye were far from statistical significance (all *p* ≥ 0.21).

### 3.2. Gender-Stratified Analysis for SNP rs112369934

To further evaluate if SNP rs112369934 was associated with any phenotypes in a gender-specific manner, we ran a statistical analysis with gender stratification. In quantitative phenotypes, we observed an association in the male POAG cases between the TC variant and both CDR and RNFL (Table 3). For CDR, there was a mean difference of −0.04 (95% CI −0.07 to −0.01) between the TT and TC genotypes with a *p*-value of 0.02 and larger CDR in TC genotypes. For RNFL, there was a mean difference of 3.73 (95% CI 0.85 to 6.61) between the TT and TC genotypes with a *p*-value of 0.011 and decreased RNFL thickness in TC genotypes. Both significant associations did not withstand Bonferroni correction for multiple comparisons. There were no associations in the quantitative phenotype analysis of female POAG cases (Table 3, all *p* ≥ 0.21).

We also observed an association in the male POAG analysis for certain qualitative phenotypes. Male POAG cases had a significant difference in abnormal optic disc size, with 0% abnormal in TC variants compared to 1.6% in TT genotypes (*p* < 0.001). TC genotypes were also associated with reduced rim depression in the interior and nasal quadrants compared to TT genotypes, with inferior depression seen in 1.2% of the TT genotypes compared to 0% in TC genotypes (*p* < 0.001) and nasal depression seen in 1.6% of the TT variants compared to 0% of TC variants (*p* < 0.001). Significant associations did not hold up to Bonferroni correction. There were no associations for qualitative phenotypes in the analysis of the female POAG cases.

## 4. Discussion

POAG is the most common form of glaucoma, a progressive and irreversible optic neuropathy that is the current leading cause of blindness globally. Here, we investigate the association of the SNP rs112369934 near the *TRIM66* gene with POAG-related qualitative and quantitative phenotypes in AAs. We found a suggestive association between male POAG cases with the TC genotype at rs112369934 and both decreased RNFL thickness and increased CDR, and no other significant associations between the SNP and phenotypic measures were found.

A previous POAAGG GWAS study implicated SNP rs112369934 on chromosome 11, near the *TRIM66* gene, as a genome-wide significant SNP associated with higher risk of POAG [34]. *TRIM66* codes for Transcriptional Intermediary Family 1-delta (Tif1-δ), a chromatin-binding protein implicated in transcriptional repression via epigenetic mechanisms [40]. At DNA double-strand breaks, Tif1-δ recruits deacetylase sirtuin-6 (SIRT6) to chromatin, which maintains DNA stability via histone deacetylation and DNA damage repair pathway activation [41]. Tif1-δ also binds heterochromatin protein 1-γ (HP1-γ), which promotes chromatin condensation for greater chromatin stability [40]. In mouse models, the differential expression patterns of *TRIM66* indicated an important role in glaucoma pathogenesis and progression to later stages of the disease [34]. Additionally, the expression of *TRIM66* is increased in induced pluripotent stem cells and human trabecular meshwork (TM) cells when subjected to oxidative stress, indicating that *TRIM66* may play a role in cellular homeostasis for retinal ganglion cells (RGCs) and TM cells under stress conditions [34].

Our study evaluated whether this variant had significant associations with quantitative and qualitative phenotypes of POAG. A prior study from our group revealed that within the POAAGG study cohort, men had a higher risk of having POAG than women [42]. In this study, we conducted gender-stratified analysis to investigate if *TRIM66* variant associations differed between men and women [42]. Although the gender-combined POAG case analysis did not reveal any significant associations, the gender-stratified analysis yielded a few interesting results. SNP rs112369934 was associated with RNFL thickness in the male POAG group, with TC genotype having decreased RNFL thickness compared to TT wildtypes, which is associated with more severe disease. The TC genotype was also associated with larger CDR in males only, compared to TT wildtype. Although these results initially suggested a significant association, they did not hold up to Bonferroni correction.

Despite falling to multiple correction, the association between rs112369934 and reduced RNFL thickness in male AA patients raises an interesting potential link between the variant and anatomic glaucomatous features. We previously reported greater prevalence of POAG in African Americans males [42]. The increased frequency of POAG in males also corresponds well with the thinner RNFL and increased CDR in males, indicating sex-related physiological differences [43]. Several members of the *TRIM* family of genes have been found to be highly expressed in the developing retina and retinal progenitor cells [35]. Our studies, as well as previous reports, implicated the protective role of *TRIM66* in oxidative stress. Differential expression due to SNP rs112369934 in *TRIM66* may affect its expression and compromise its protective role in RGCs in males when compared to females. Establishing the regulatory role of SNP rs31732228 with regards to sex-based differences warrants further investigation. Structural changes to the RNFL are characteristic of glaucomatous changes to the posterior eye. Previous studies have shown that glaucoma suspects with visual field defects have nearly double the rate of RNFL thinning, representing global ganglion nerve cell death [44,45]. As the RNFL thins due to RGC death, the disc that represents the converging nerve fibers decreases, and the cup that represents the negative space in the optic channel enlarges, increasing the CDR [46]. However, the nature of CDR assessment makes CDR an insensitive method for evaluating ganglion cell loss due to glaucomatous changes, as miniscule changes to CDR may represent large losses of RGCs, especially in patients with large CDRs at baseline [46]. Our results may suggest that there is a link between the SNP and glaucomatous changes such as increased ganglion cell death, leading to decreased RNFL thickness and increased CDR.

The additional gender-stratified analysis of qualitative phenotypes suggested a potential association with TC genotypes having less abnormal disc sizes and reduced inferior and nasal rim depression compared to wildtype TT genotypes. Despite the suggestive *p*-values, there is missing data for qualitative phenotypes, as observed in Table 3, due to a combination of missing images for grading along with poor image quality. There are also no data values for TC variants in these phenotypes, which could be a reflection of inadequate sample size due to low TC genotype prevalence in the study population. Since African Americans have greater genetic variation than European Americans [47], a larger cohort may be needed to detect significant SNP effects in GWAS analysis. Future studies with large sample size are needed to properly fit the statistical model employed as described above and produce reliable results.

As mentioned, this study hints at the role of SNP rs112369934 in POAG pathogenesis via RGC death. The potential mechanisms are varied given the many roles of *TRIM66* in activating DNA damage repair pathways, stabilizing chromatin, and protecting against oxidative stress. Given that SNP rs112369934 is associated with higher POAG risk in both males and females [34] but is only found to have suggestive associations with phenotypes in male POAG cases, there may be a larger functional role for rs112369934 beyond anatomic characteristics that contributes to POAG pathogenesis. Future studies are underway to assess the functional role of rs112369934 via in vitro expression studies and knockout studies in the induced pluripotent stem-cell-derived retinal ganglion cell cultures (iPSC-RGCs) established by our lab [48].

This study continues the work of the POAAGG study, a critically important project that aims to identify significant variants in the Philadelphian AA population. By identifying more genetic variants associated with POAG pathogenesis, this work will allow for a greater understanding of the heterogenous nature of glaucoma presentation and increase the likelihood for early disease detection, early intervention, and identification of therapeutic targets to help prevent irreversible blindness in AAs.

## Figures and Tables

**Table 1:** Association of *TRIM66* rs112369934 in quantitative phenotypes.

Phenotype	Wildtype TT		Variant TC		Mean Difference (95% CI)	*p*-Value
	N Eyes	Mean (SD)	N Eyes	Mean (SD)		
MD (dB)	3400	−8.36 (9.02)	457	−8.64 (9.04)	0.27 (−0.81 to 1.36)	0.62
PSD (dB)	3403	5.20 (3.47)	457	5.27 (3.40)	−0.07 (−0.45 to 0.32)	0.73
CCT	3961	533.84 (40.07)	578	529.61 (36.46)	4.23 (−0.09 to 8.55)	0.055
CDR	3986	0.71 (0.17)	569	0.72 (0.17)	−0.02 (−0.03 to 0.00)	0.12
IOP	4241	17.32 (6.00)	617	17.67 (6.66)	−0.35 (−1.06 to 0.35)	0.33
VA.CC	3653	0.38 (0.84)	522	0.46 (0.92)	−0.08 (−0.17 to 0.01)	0.09
VA.SC	2791	0.57 (0.98)	374	0.69 (1.12)	−0.12 (−0.26 to 0.02)	0.09
RNFL	3401	73.40 (14.98)	475	72.29 (15.50)	1.12 (−0.73 to 2.96)	0.24

Mean deviation (MD), pattern standard deviation (PSD), central corneal thickness (CCT), cup-to-disc ratio (CDR), intra-ocular pressure (IOP), visual acuity (VA) with correction (CC) and without correction (SC), retinal nerve fiber layer (RNFL).

**Table 2:** Association of *TRIM66* rs112369934 in qualitative POAG phenotypes ^1^.

Phenotype	Wildtype	Variant	*p*-Value
	TT (N = 3227 Eyes)	TC (N = 462 Eyes)	
Disc Shape			0.53
Round	944 (48.4%)	120 (46.0%)	
Oval	1008 (51.6%)	141 (54.0%)	
Disc Size			0.61
Normal	1934 (98.6%)	259 (98.1%)	
Abnormal	27 (1.4%)	5 (1.9%)	
Shape of Cup			0.34
Conical	691 (37.0%)	93 (37.8%)	
Cylindrical	934 (50.0%)	112 (45.5%)	
Bean	243 (13.0%)	41 (16.7%)	
Cup Depth			0.39
Shallow	250 (13.3%)	25 (10.2%)	
Moderate	1193 (63.3%)	165 (67.1%)	
Severe	441 (23.4%)	56 (22.8%)	
Rim Plane Position Constant			0.54
No	288 (15.3%)	42 (17.0%)	
Yes	1598 (84.7%)	205 (83.0%)	
Rim Depressed Inferior			0.95
No	1864 (98.8%)	244 (98.8%)	
Yes	22 (1.2%)	3 (1.2%)	
Rim Depressed Superior			NA
No	1886 (100.0%)	247 (100.0%)	
Yes	0 (0.0%)	0 (0.0%)	
Rim Depressed Nasal			0.31
No	1854 (98.3%)	245 (99.2%)	
Yes	32 (1.7%)	2 (0.8%)	
Rim Depressed Temporal			0.37
No	1640 (87.0%)	209 (84.6%)	
Yes	246 (13.0%)	38 (15.4%)	
Presence of PPA			NA
No	0 (0.0%)	0 (0.0%)	
Yes	1946 (100.0%)	262 (100.0%)	
Borders of PPA			0.68
Indistinct	243 (12.5%)	30 (11.5%)	
Distinct	1701 (87.5%)	231 (88.5%)	
Excavation			0.37
No	1415 (74.9%)	194 (77.9%)	
Yes	473 (25.1%)	55 (22.1%)	
Heavy TM Pigmentation			0.33
Indistinct	1499 (77.1%)	192 (73.8%)	
Distinct	446 (22.9%)	68 (26.2%)	
Disc Tilt			0.08
No	1841 (94.6%)	237 (91.2%)	
Yes	106 (5.4%)	23 (8.8%)	
Disc Hemorrhage			0.41
No	1914 (98.2%)	255 (97.3%)	
Yes	36 (1.8%)	7 (2.7%)	
Notching			0.21
No	1797 (93.9%)	244 (96.1%)	
Yes	116 (6.1%)	10 (3.9%)	
Disc Pallor			0.69
No	1886 (96.7%)	253 (96.2%)	
Yes	64 (3.3%)	10 (3.8%)	

^1^ A portion of images for qualitative phenotypic grading were missing or of poor quality, leading to missing data for the qualitative phenotypes for both genotypes.

**Table 3:** Association of *TRIM66* rs112369934 in our measured quantitative phenotypes in gender stratified analysis.

Gender	Phenotype	Wildtype TT		Variant TC		Mean Difference (95% CI)	*p*-Value
		N Eyes	Mean (SD)	N Eyes	Mean (SD)		
Males	MD (dB)	1492	−9.38 (9.89)	158	−10.83 (10.71)	1.45 (−0.63 to 3.54)	0.17
	PSD (dB)	1492	5.33 (3.51)	158	5.60 (3.50)	−0.27 (−0.87 to 0.34)	0.39
	CCT	1680	534.78 (40.80)	206	529.12 (34.40)	5.66 (−1.27 to 12.59)	0.11
	CDR	1737	0.73 (0.17)	210	0.77 (0.17)	−0.04 (−0.07 to −0.01)	0.02
	IOP	1824	17.27 (6.17)	223	17.97 (7.82)	−0.70 (−2.07 to 0.67)	0.32
	VA.CC	1548	0.43 (0.93)	183	0.53 (0.94)	−0.09 (−0.25 to 0.06)	0.23
	VA.SC	1204	0.63 (1.09)	147	0.73 (1.10)	−0.10 (−0.32 to 0.12)	0.38
	RNFL	1438	71.47 (14.84)	160	67.74 (14.88)	3.73 (0.85 to 6.61)	0.01
Females	MD (dB)	1908	−7.57 (8.19)	299	−7.48 (7.78)	−0.09 (−1.29 to 1.11)	0.88
	PSD (dB)	1911	5.10 (3.44)	299	5.09 (3.34)	0.00 (−0.49 to 0.50)	0.99
	CCT	2281	533.15 (39.52)	372	529.89 (37.60)	3.27 (−2.25 to 8.78)	0.25
	CDR	2249	0.69 (0.17)	359	0.70 (0.16)	−0.01 (−0.03 to 0.02)	0.56
	IOP	2417	17.35 (5.87)	394	17.50 (5.91)	−0.15 (−0.94 to 0.64)	0.72
	VA.CC	2105	0.35 (0.76)	339	0.43 (0.91)	−0.08 (−0.19 to 0.03)	0.16
	VA.SC	1587	0.52 (0.88)	227	0.66 (1.14)	−0.14 (−0.31 to 0.04)	0.12
	RNFL	1963	74.82 (14.92)	315	74.59 (15.33)	0.22 (−2.07 to 2.52)	0.85

Mean deviation (MD), pattern standard deviation (PSD), central corneal thickness (CCT), cup-to-disc ratio (CDR), intra-ocular pressure (IOP), visual acuity (VA) with correction (CC) and without correction (SC), retinal nerve fiber layer (RNFL).

## Data Availability

All POAAGG genotype files are available from the dbGap database (accession number phs001312.v1.p1; URL: https://www.ncbi.nlm.nih.gov/projects/gap/cgi-bin/study.cgi?study_id=phs001312.v1.p1) (accessed on 1 July 2021).
